# Development of a multivariable prognostic PREdiction model for 1-year risk of FALLing in a cohort of community-dwelling older adults aged 75 years and above (PREFALL)

**DOI:** 10.1186/s12877-021-02346-z

**Published:** 2021-06-30

**Authors:** Gustav Valentin Gade, Martin G. Jørgensen, Jesper Ryg, Tahir Masud, Lasse Hjort Jakobsen, Stig Andersen

**Affiliations:** 1 Department of Geriatric Medicine, Aalborg University Hospital, Hobrovej 18-22, Gl. Roed Bygn. 6, 9000 Aalborg, Denmark; 2 Department of Clinical Medicine, Aalborg University, Soendre Skovvej 15, 9000 Aalborg, Denmark; 3 Department of Geriatric Medicine, Odense University Hospital, Sdr. Boulevard 29, 5000 Odense, Denmark; 4 Department of Clinical Research, University of Southern Denmark, Winsloewparken 19, 5000 Odense, Denmark; 5 Department of Healthcare for Older People, Nottingham University Hospitals NHS Trust, Derby Road, Nottingham, Nottinghamshire, NG7 2UH UK; 6 Department of Hematology, Aalborg University Hospital, Moelleparkvej 4, Aalborg, Denmark

**Keywords:** Accidental falls, Models, Theoretical, Multivariable analysis, Prognosis

## Abstract

**Background:**

Falls are the leading cause of fatal and non-fatal injuries in older adults, and attention to falls prevention is imperative. Prognostic models identifying high-risk individuals could guide fall-preventive interventions in the rapidly growing older population. We aimed to develop a prognostic prediction model on falls rate in community-dwelling older adults.

**Methods:**

Design: prospective cohort study with 12 months follow-up and participants recruited from June 14, 2018, to July 18, 2019.

Setting: general population.

Subjects: community-dwelling older adults aged 75+ years, without dementia or acute illness, and able to stand unsupported for one minute.

Outcome: fall rate for 12 months.

Statistical methods: candidate predictors were physical and cognitive tests along with self-report questionnaires. We developed a Poisson model using least absolute shrinkage and selection operator penalization, leave-one-out cross-validation, and bootstrap resampling with 1000 iterations.

**Results:**

Sample size at study start and end was 241 and 198 (82%), respectively. The number of fallers was 87 (36%), and the fall rate was 0.94 falls per person-year. Predictors included in the final model were educational level, dizziness, alcohol consumption, prior falls, self-perceived falls risk, disability, and depressive symptoms. Mean absolute error (95% CI) was 0.88 falls (0.71–1.16).

**Conclusion:**

We developed a falls prediction model for community-dwelling older adults in a general population setting. The model was developed by selecting predictors from among physical and cognitive tests along with self-report questionnaires. The final model included only the questionnaire-based predictors, and its predictions had an average imprecision of less than one fall, thereby making it appropriate for clinical practice. Future external validation is needed.

**Trial registration:**

Clinicaltrials.gov (NCT03608709).

**Supplementary Information:**

The online version contains supplementary material available at 10.1186/s12877-021-02346-z.

## Background

Fall accidents are frequent in community-dwelling older adults. Around 30% of all 65 + −year olds fall yearly. This increases to 50% in older adults aged 80 years or above [1]. With the current demographic trends, the proportion of older adults will increase [1], and consequently, the number of people falling will rise. Falls are associated with increased morbidity and mortality along with loss of independence, which ultimately may lead to earlier placement in long-term care [2–4]. Furthermore, health care costs related to falls increase with age [5]. Therefore, early falls prevention is imperative and may be most beneficial to individuals at high risk of falling [6].

Identifying high-risk individuals is not straightforward due to the multifactorial and complex nature of falls with more than 400 acknowledged risk factors [4, 7]. Furthermore, these associations refer to group differences on specific risk factors. In contrast, personal predictions are estimates of absolute risks on an individual level [8]. Prognostic models predict an individual’s risk of a future outcome and are typically developed by evaluating two or more risk factors in combination [9]. Thus, older adults may have their risk of falling estimated to better target interventions. A recently published systematic review [10] highlighted models available for predicting falls [11–17]. These models were primarily based on younger participants (65+ years) [11–13, 15–17]. Also, methodological limitations were present regarding outcome assessments with long recording intervals and lack of blinding [11–17] which could potentially lead to unreliable predictions. Moreover, to support the implementation of models, applicability in clinical practice is a vital issue to address [18].

This study aimed to develop a multifactorial prognostic prediction model for estimating the risk of falling in community-dwelling older adults.

## Methods

This prospective cohort study recruited participants from 14/06/2018 to 18/07/2019, with a 12-month follow-up period. A pre-registered protocol was submitted at Clinicaltrials.gov (NCT03608709) [19] on 01/08/2018. The reporting of the study followed the Transparent Reporting of a multivariable prediction model for Individual Prognosis Or Diagnosis (TRIPOD) statement [20] and Strengthening the Reporting of OBservational studies in Epidemiology (STROBE) statement [21]. TRIPOD and STROBE checklists are available in Additional file 1: appendix 1. To adhere fully to the reporting guidelines, a more detailed description of the population, recruitment sites, study dates, data collection procedures on predictors and outcomes, along with statistical analyses and results are available in Additional file 1: appendix 2.

### Sources of data

This was a study conducted in a general population setting within a single municipality.

### Participants

Community-dwellers aged 75 years or above residing in Hjørring Municipality, Northern Jutland, Denmark, were studied. We recruited participants through preventive home visits (PHV), senior activity centers (SAC), along with senior clubs and associations. Participants were assessed for eligibility and included consecutively in PHVs and by convenience in the remaining recruitment sites. Exclusion criteria were living in care facilities, the presence of self-reported acute illness within seven days before recruitment, being unable to stand for one minute without any assistive device or support from another person, unable to understand Danish, or having a dementia diagnosis. The latter was checked through hospital records with informed consent from participants. Data collectors only recorded whether the older adults were eligible for participation or not due to time constraints.

### Outcome

We defined falls as “an unexpected event in which the participants come to rest on the ground floor or lower level” [22]. The primary outcome for prediction was falls rate within 12 months. Fall calendars with a daily recording of falls were returned monthly by post. If calendars had falls recorded, research secretaries contacted participants by telephone to validate and describe the falls.

### Predictors

The test battery has been described previously [23]. In brief, it consisted of physical tests of static balance under dual-tasking conditions, grip- and lower limb strength, reaction time of lower limbs, and habitual gait speed; a self-report questionnaire on demographic characteristics, frailty, nutrition, disability, fear of falling, health-related quality of life, depression, several physical symptoms, and a cognitive test. Static balance, reaction time, grip- and lower limb strength were measured using a Nintendo Wii Balance Board due to its valid and reliable measures and portable setup [24–27]. Gait speed was measured using the four-meter gait speed test [28]. Furthermore, frailty was assessed using Tilburg Frailty Indicator (TFI) [29], nutrition using the short Mini Nutritional Assessment (MNA) [30], disability using Vulnerable Elders Survey 13, (VES) [31], fear of falling using short Falls Efficacy Scale International [32], health-related quality of life using EuroQol Five Dimensions Three Levels (EQ-5D-3L), depressive symptoms using the Geriatric Depression Scale with four items (GDS) [33], and the cognitive test using the Orientation-Memory-Concentration test [34]. In total, 34 candidate predictors corresponding to 41 degrees of freedom were available for model building. Additional file 1: Appendix 3. eTable 1 provides details of all candidate predictors, how they were defined, along with how and when they were measured.

### Blinding

Municipality staff and the first author performed the baseline data collection. Data collectors in PHVs and SACs were blinded to all predictors except the physical tests. Participants were blinded to all predictors except those in the questionnaire and the number of all prescribed drugs. The first author was blinded to physical tests performed in PHVs and SACs. Research secretaries assessing the outcome were blinded to baseline predictors.

### Statistical analyses

A recommended method for calculating sample size was not available when the study commenced. Thus, we based the sample size on feasible recruitment within the 13 months inclusion period granted by the municipality.

#### Descriptive statistics and comparisons

We summarized continuous variables using medians with interquartile range. Categorical variables were summarized using proportions. We compared baseline demographics between recruitment sites and follow-up completeness (Additional file 1: appendix 2). Finally, we calculated univariate falls incidence rate ratios (IRR) for all predictors using Poisson regression.

#### Missing data

We summed the number of missing values per predictor and the number of subjects with missing data. We compared participants with and without missing data similarly to follow-up completeness comparison (see Additional file 1: appendix 2). Missing data were imputed by a single imputation in a random forest imputation scheme to avoid excessive computation time associated with multiple imputation techniques and preserve imputations’ precision [35]. However, we chose a mode imputation scheme for categorical variables with less than ten observations in a category. The imputation procedures used all candidate predictors without missing data (see Additonal file 1: appendix 3 – eTable 1).

#### Handling of predictors in the analyses

All continuous variables were kept continuous and modeled linearly to avoid loss of information by dichotomization [36]. Variables used for generating a score, e.g., the Tilburg Frailty Indicator, were not included in the modeling process; we only included the scores. Scores were generated after imputing missing data. For categorical variables, all cut points were pre-specified and summarized in Additional file 1: appendix 3. eTable 1.

#### Modeling

Follow-up time was defined as the time from inclusion until death, loss to follow-up, or end of the study. Fall rate was calculated as the total number of falls divided by length of follow-up in years. The fall rate was modeled using a Poisson regression model with log-follow-up time as off-set and number of falls as the dependent variable. We fitted the model using least absolute shrinkage and selection operator penalization [37] with leave-one-out cross-validation to perform variables selection and address overfitting during modeling. Thus, the abovementioned univariate analyses did not guide predictor selection in the modeling process. Interaction terms were not tested due to limited sample size. Furthermore, to guide the prediction modeling process, we pre-specified the expected direction of predictor effects (Additional file 1: appendix 3 – eTable 1). Performance measures were internally validated using a bootstrap resampling technique with 1000 samples drawn with replacement from the original sample. The model was fitted on the bootstrap sample and validated on the out-of-bag sample. Participants who died or were lost to follow-up were always included in the bootstrap sample as the true number of falls was not observed in these participants. To quantify model performance, we computed the mean absolute difference between each participant’s predicted and observed number of falls. This is also known as the mean absolute error (MAE) and reflects how imprecisely the model predicts on the entire sample using the unit: number of falls. We derived a 95% confidence interval from the 2.5th and 97.5th percentiles of the 1000 MAEs from the bootstrap resampling procedure. Lastly, we also calculated the mean squared error (MSE) with a 95% confidence interval.

#### Software

All statistical analyses were performed using R, version 4.0.2, (R Foundation) with packages: mlr, glmnet, pseudo, pROC, and reshape2.

## Results

### Descriptive characteristics

**Fig. 1:**
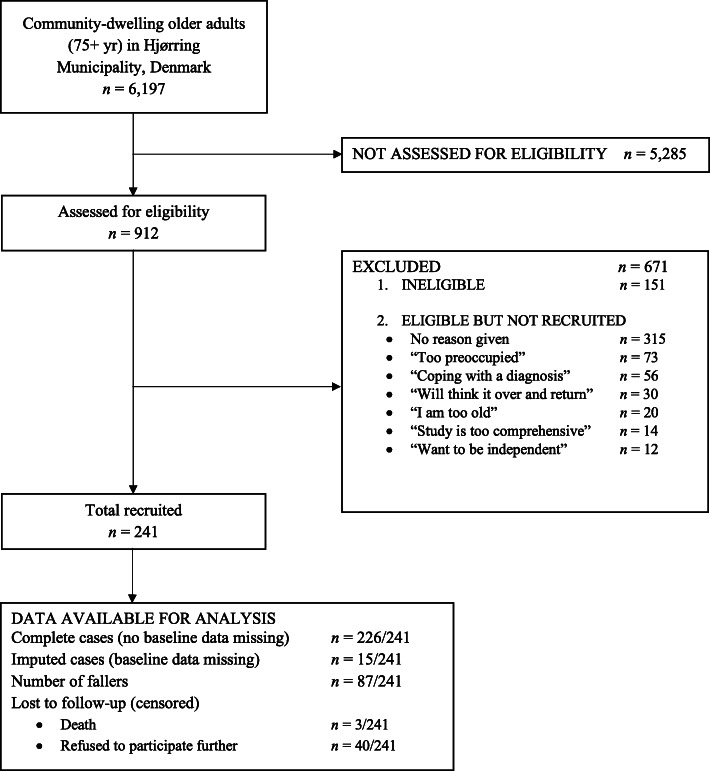
Flow diagram displaying participants at each stage in the study

During the study period, 6197 community-dwelling older adults (75+ years) lived in the municipality. Of these, we assessed 912 for eligibility, from which we included 241 older adults (66.4% women) with median (IQR) age 82 (80; 86) years. Figure 1 displays the flow of participants, and Table 1 provides the baseline characteristics of participants. Fifteen (6%) participants had missing data on predictors. Their demographic characteristics did not differ from participants without missing data.

**Table 1:** Cohort baseline characteristics

Variable	Level and summary measure	Total ^a^
**Sociodemographics**		
Age (years)	median [IQR]	82 [80, 86]
Sex	Women, n (%)	160 (66.4)
Marital status	Married or living with a partner, n (%)	98 (40.7)
	Widow, n (%)	125 (51.9)
	Other, n (%)	18 (7.5)
Living alone	Yes, n (%)	144 (59.8)
Highest level of education completed	Municipal primary and lower secondary school, n (%)	106 (44.0)
	Skilled worker, n (%)	68 (28.2)
	General upper secondary education or longer, n (%)	67 (27.8)
Monthly household income in Euro	< 1075, n (%)	106 (44.0)
	1076 - 2285, n (%)	81 (33.6)
	> 2285, n (%)	54 (22.4)
Weekly alcohol consumption (units)	< 7, n (%)	187 (77.6)
	7–14, n (%)	47 (19.5)
	> 14, n (%)	7 (2.9)
Having dog or cats in the household	Yes, n (%)	30 (12.4)
Using glasses with multifocal lenses	Yes, n (%)	184 (76.3)
**Falls**		
One or more falls within the previous year	Yes, n (%)	105 (43.6)
Do you think you will fall within the next year?	Yes, n (%)	45 (18.7)
Short Falls Efficacy Scale International (7–28) #	median [IQR]	8 [7, 11]
**Comorbidities and medication**		
Having two or more diseases or chronic disorders	Yes, n (%)	209 (86.7)
Charlson Comorbidity Index - score (0–37) #	median [IQR]	1 [0, 2]
Charlson Comorbidity Index – grouping #	0, n (%)	106 (44.0)
	1–2, n (%)	108 (44.8)
	> 3, n (%)	27 (11.2)
Number of all prescribed drugs	median [IQR]	7 [4, 10]
Polypharmacy (5 or more prescribed drugs)	Yes, n (%)	170 (70.5)
**Frailty and symptoms**		
Tilburg Frailty Indicator - Total score (0–15) #	median [IQR]	6 [4, 8] ^b^
Occasionally experiencing dizziness	Yes, n (%)	123 (51.0)
Occasionally experiencing lower limb pain when walking?	Yes, n (%)	121 (50.2)
Occasionally experiencing urinary incontinence	Yes, n (%)	90 (37.3)
**Nutrition**		
Mini Nutritional Assessment Score (0–14)	median [IQR]	11 [10, 12] ^b^
Body Mass Index (kg/m^2^)	median [IQR]	27.7 [25.3,30.7] ^b^
**Psychology and cognition**		
Geriatric Depression Scale - 4 items #	Two or more points, n (%)	13 (5.4)
Orientation-Memory-Concentration test (0–28)	median [IQR]	26 [22, 26] ^d^
EQ-5D-3L score (−0.167–1.000)	median [IQR]	0.7 [0.6, 1.0]
**Activities of Daily Living and mobility**		
Vulnerable Elders Survey - 13 (1–10) #	median [IQR]	3 [1, 4] ^d^
Using walking aids	Yes, n (%)	142 (58.9)
Habitual gait speed (m/sec)	median [IQR]	0.9 [0.7, 1.2]
**Physical**		
Total grip strength of both upper limbs (kg)	median [IQR]	24.3 [18.6, 30.2] ^c^
Difference in grip strength between upper limbs (kg)	median [IQR]	1.5 [0.7, 2.5] ^c^
Total isometric lower limb strength (kg)	median [IQR]	132.8 [90.1, 188.4] ^c^
Difference in isometric lower limb strengths (kg)	median [IQR]	7.1 [3.4, 11.9] ^c^
Average lower limb reaction time (ms)	median [IQR]	1315 [1122; 1567] ^c^
Difference in lower limb reaction time (ms)	median [IQR]	142 [60, 370] ^c^
Centre of Pressure area (mm^2^)	median [IQR]	69 [42, 112] ^b^
Centre of Pressure speed (mm/s)	median [IQR]	23 [16, 30] ^b^

*Notes: n* number, % percentage proportion, *IQR* interquartile range, # a lower score is better, *m/sec* meters per second, *kg* kilogram, *ms* milliseconds, *mm*^*2*^ square millimeters, *mm/s* millimeters per second, *kg/m*^*2*^ kg per square meters, ^a^ = sample size is 241 participants, ^b^ = 1 missing observation, ^c^ = 2 missing observations, ^d^ = 4 missing observations

### Outcome

The median (IQR) follow-up time for all participants was 365 (365–365) days. Forty-three (18%) participants had an incomplete follow-up. Median (IQR) age was higher for these, 85 (81–87) years, compared to participants retained in the study, 82 (79–86) years, (*p* = 0.01). Eighty-seven (36%) participants fell during follow-up, and 44 of these fell more than once. In total, we recorded 178 falls and calculated a fall rate of 0.94 falls per person-year. Table 2 displays univariate falls IRR for all predictors. Risk factors associated with falls were the completion of a short-cycle or medium-cycle higher education (IRR 2.97, 95% CI 1.80–4.74; IRR 2.00, 95% CI 1.36–2.92), drinking more than seven units of alcohol weekly (IRR 1.55, 95% CI 1.12–2.11), using walking aids (IRR 1.37, 95% CI 1.02–1.83), having three points on the Geriatric Depression Scale (IRR 9.5, 95% CI 5.34–15.70), having fallen within the last year (IRR 3.47, 95% CI 2.53–4.85), believing to fall within the next year (IRR 4.10, 95% CI 3.04–5.50), presence of dizziness (IRR 2.17, 95% CI 1.59–2.99), and increased balance sway area when dual-tasking (IRR 1.00, 95% CI 1.00–1.00). An increasing number of prescribed drugs (IRR 1.06, 95% CI 1.02–1.10), TFI score (IRR 1.13, 95% CI 1.06–1.20), Short Falls Efficacy Scale score (IRR 1.06, 95% CI 1.01–1.11), and VES score (IRR 1.13, 95% CI 1.07–1.18) were also associated with falls. Finally, use of multifocal lenses (IRR 0.60, 95% CI 0.44–0.83) along with increasing age (IRR 0.97, 95% CI 0.94–1.00), MNA score (IRR 0.84, 95% CI 0.78–0.91), and EQ-5D-3L score (IRR 0.19, 95% CI 0.09–0.40) were associated with a lower falls rate.

**Table 2:** Univariate falls incidence rate ratios for characteristics of 241 community-dwelling older adults (75+ years)

Variable	Unit or category	IRR (95% CI)	***p***-value
**Sociodemographics**			
Age (years)	Years	0.97 (0.94–1.00)	**<.05**
Sex	Men	*Ref*	*Ref*
	Women	0.90 (0.67–1.23)	.52
Marital status	Married or living with a partner	*Ref*	*Ref*
	Not married	1.44 (0.60–2.90)	.36
	Separated or divorced	0.91 (0.35–1.93)	.83
	Widow	1.08 (0.79–1.47)	.64
Living alone	No	*Ref*	*Ref*
	Yes	1.19 (0.88–1.61)	.27
Highest level of education completed	Municipal primary and lower secondary school	*Ref*	*Ref*
	General upper secondary education	0.76 (0.19–0.21)	.64
	Skilled worker	1.01 (0.67–1.49)	.97
	Short-cycle higher education	2.97 (1.80–4.74)	**< .05**
	Medium-cycle higher education	2.00 (1.36–2.92)	**< .05**
	Long cycle higher education	0.84 (0.25–2.03)	.73
Weekly alcohol consumption	0–7 units	*Ref*	*Ref*
	> 7 units	1.55 (1.12–2.11)	**<.05**
Dogs or cats in the household	No	*Ref*	*Ref*
	Yes	1.26 (0.82–1.85)	.27
Using glasses with multifocal lenses	No	*Ref*	*Ref*
	Yes	0.60 (0.44–0.83)	**<.05**
**Comorbidities and medication**			
Having two or more diseases or chronic disorders	No	*Ref*	*Ref*
	Yes	1.35 (0.86–2.25)	.21
Number of all prescribed drugs	Count	1.06 (1.02–1.10)	**<.05**
**Nutrition**			
Body Mass Index	Kg/m^2^	0.98 (0.95–1.01) ^a^	.24
Mini Nutritional Assessment Score	Score from 0 to 14 (14 is the best possible score)	0.84 (0.78–0.91) ^a^	**<.05**
**Activities of Daily Living and mobility**			
Vulnerable Elders Survey – 13	Score from 1 to 10 (1 is the best score)	1.13 (1.07–1.18)	**<.05**
Using walking aids	No	*Ref*	*Ref*
	Yes	1.37 (1.02–1.83)	**<.05**
Habitual gait speed	M/sec	0.88 (0.65–1.18)	.41
**Psychology and cognition**			
Geriatric Depression Scale - 4 items	0	*Ref*	*Ref*
	1	0.76 (0.50–1.13)	.20
	2	1.15 (0.54–2.14)	.67
	3	9.5 (5.34–15.70)	**<.05**
Orientation-Memory-Concentration test	Score from 0 to 28 (28 is the best score)	0.99 (0.96–1.03) ^c^	.69
EQ-5D-3L	Score from −0.167 to 1.000 points	0.19 (0.09–0.40)	**<.05**
**Falls**			
One or more falls within the previous year	No	*Ref*	*Ref*
	Yes	3.47 (2.53–4.85)	**<.05**
Short Falls Efficacy Scale International	Score from 7 to 28 (7 is the best score)	1.06 (1.01–1.11)	**<.05**
Do you think that you will fall within the next year?	No	*Ref*	*Ref*
	Yes	4.10 (3.04–5.50) ^b^	**<.05**
**Frailty and symptoms**			
Tilburg Frailty Indicator	Score from 0 to 15 points (0 is the best possible score)	1.13 (1.06–1.20) ^a^	**<.05**
Occasionally experiencing dizziness	No	*Ref*	*Ref*
	Yes	2.17 (1.59–2.99)	**<.05**
Occasionally experiencing lower limb pain when walking?	No	*Ref*	*Ref*
	Yes	1.29 (0.96–1.74)	.09
Occasionally experiencing urinary incontinence	No	*Ref*	*Ref*
	Yes	1.25 (0.92–1.67)	.15
**Physical**			
Total grip strength of both upper limbs	Kg	0.99 (0.97–1.00) ^b^	.17
Difference in grip strength between upper limbs	Kg	0.95 (0.86–1.04) ^b^	.34
Total isometric lower limb strength	Kg	0.98 (0.96–1.00) ^b, d^	.08
Difference in isometric lower limb strengths	Kg	0.78 (0.62–0.97) ^b, d^	**.03**
Average lower limb reaction time	Ms	0.99 (0.99–1.00) ^b, d^	.96
Difference in lower limb reaction time	Ms	0.99 (0.99–1.00) ^b, e^	.09
Centre of Pressure area	mm^2^	1.01 (1.00–1.02) ^a, d^	**<.05**
Centre of Pressure speed	mm/s	1.04 (0.94–1.14) ^a, d^	.45

*Notes: n* number, % percentage proportion, *IRR* incidence rate ratio, *CI* confidence interval, *Ref* reference group, *m/sec* meters per second, *kg* kilogram, *ms* milliseconds, *mm*^*2*^ square millimeters, *mm/s* millimeters per second, *kg/m*^*2*^ kg per square meters, ^a^ = 1 missing observation, ^b^ = 2 missing observations, ^c^ = 4 missing observations, ^d^ = data has been scaled by a factor of 10, ^e^ = IRR (95% CI): 0.9997914 (0.9995242–1.000003)

### Prediction modeling

**Fig. 2:**
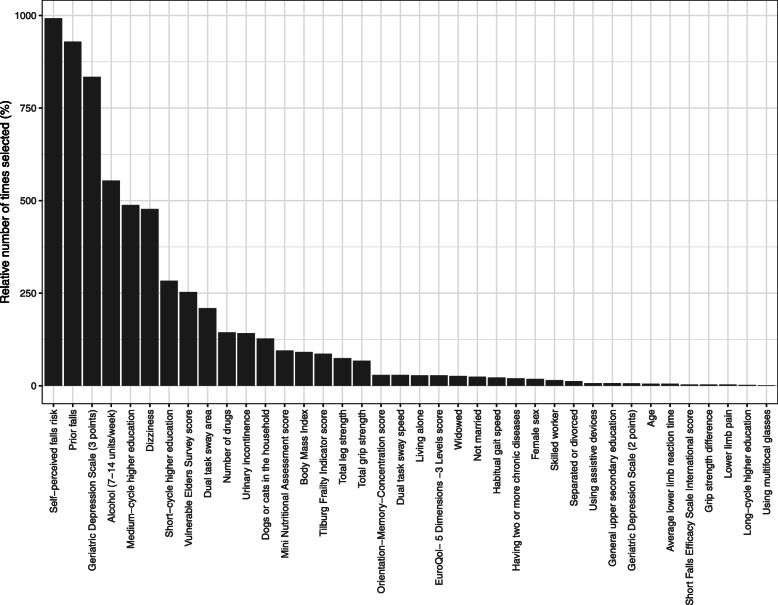
Predictors most frequently selected for prediction models in 1000 bootstraps

Figure 2 displays which predictors were most frequently selected for the prediction model in each bootstrap. Table 3 gives the fully specified prediction model and its performance measures. The final model included educational level, dizziness, weekly alcohol consumption, prior falls, self-perceived falls risk, disability, and depressive symptoms; all reported in a 22-item self-report questionnaire. For apparent performance, i.e., before internal validation, the MAE was 0.80 falls. After internal validation, MAE (95% CI) increased to 0.88 (0.71–1.16) falls. Additional file 1: Appendix 4. eTable 2 gives an example of how to use the model.

**Table 3:** Final prediction model on fall rate and performance measures

Sample size (n)	241
Number of events (falls)	178
**Intercept and predictors**	**Beta coefficient**
Intercept	−6.89417948
Short-cycle higher education (completed)	0.14752490
Medium-cycle higher education	0.22365429
Occasional dizziness (present)	0.17787573
Weekly alcohol consumption: 7–14 units (yes)	0.28918905
Fallen within the last year (yes)	0.50322396
Self-perceived falls risk (will fall)	0.74611041
VES score	0.01768701
3 points on GDS	1.21255321
**Performance**	
Apparent mean squared error	1.11
Bootstrapped mean squared error (95% CI)	1.92 (0.81–5.10)
Apparent mean absolute error	0.80
Bootstrapped mean absolute error (95% CI)	0.88 (0.71–1.16)

*Notes: VES score* Vulnerable Elders Survey 13 score, *GDS* Geriatric Depression Scale with 4 items, *kg* kilogram, *CI* Confidence interval

## Discussion

This study aimed to develop a falls prediction model for community-dwelling older adults. Only self-reported predictors were included in the final model. This makes it a simple and clinically applicable model. The model predictions had an average imprecision of less than one fall.

### Comparison to other studies

Other studies have developed falls prediction models for community-dwelling older adults with the same setting and falls definition as in the present study [11–17]. Models were either purely questionnaire-based [13, 15, 17] or a combination of questionnaires and objective measures [11, 12, 15, 16]. Similarly, they found prior falls [12, 13, 15–17], self-perceived falls risk [17], activities of daily living [12, 16], dizziness [12, 16], educational level [12, 16], alcohol consumption [12, 16], and depressive symptoms [11] to predict falls. In contrast to these studies, we did not find self-reported visual impairment [11, 13, 15], grip strength [12, 16], body mass index [11, 12], medication [13], or global cognition [11] to be predictive of falls. This discrepancy may be due to different participant characteristics along with assessment methods for outcomes and predictors. For participant characteristics, younger participants (> 65 years) were included [11–13, 15–17] resulting in a median age around 75 years for the majority of studies [11–13, 16]. In contrast, our sample was older but smaller. Thus, our composition of predictors may only be representative for older adults (75+ years). For outcome assessments, we applied fall calendars, with a daily recording of falls, returned monthly, and outcome assessors blinded to baseline predictors. For the other studies, falls were recorded either yearly [15], quarterly [13, 16, 17], or weekly [11, 12, 14] which may increase the risk of recall bias and influence predictor selection for models. Also, only one study reported whether outcome assessors were blinded when validating falls according to their definition [17]. Thus, it is unclear whether detection bias influencing the predictive abilities of risk factors was present in the remaining studies. Lastly, differing measurement methods may cause different predictive abilities for the same risk factor across studies. These limitations can potentially lead to the inclusion of risk factors with poor predictive abilities in models. Consequently, model predictors may differ, and predictions may be less reliable when using models in clinical practice.

### Implications for clinical practice

On a group level, we found 16 risk factors associated with falls through univariate analyses. However, only half of these were able to predict falls on an individual level. This emphasizes the need for clinicians to know when to best apply evidence from association and prediction modeling studies on falls since a statistically significant association does not imply that a risk factor is a strong predictor. For example, lower limb strength is strongly associated with falls [38] but may be less predictive of falls in community-dwelling older adults in the general population [12, 15, 16]. In our study, lower limb strength was not included in our prediction model, indicating no additional predictive value in the presence of the other candidate predictors. This was substantiated during our model development, where lower limb strength was less frequently chosen than the predictors in the final model. This finding is in line with three other prediction modeling studies measuring lower limb strength [12, 15, 16]. Here, only one study included a measure of lower limb strength in a final model but concluded that this was not superior to simpler questionnaire-based models [15]. Reasons for this could be because the test does not differentiate well between fallers and non-fallers as the average lower limb strength compares to the background population [39], and more functional tests of lower limb strength could be more relevant in terms of investigating for predictive performance. This knowledge may be applied when following the current recommendations for falls prevention [6]. Here, initial screening of falls risk is necessary for referring high-risk individuals to further assessment for modifiable risk factors and targeted interventions. Falls prediction models are appropriate for screening but not necessarily explaining falls risk. When presumed high-risk individuals have been identified, evidence on associated modifiable falls risk factors can explain why the older person is at risk and what to intervene on. In this scenario, self-report questionnaire models that predict falls may be both time-efficient and implementable due to their simplicity. Regarding time consumption, our model included the Vulnerable Elders Survey 13 [31], taking five minutes to administer [40], and nine questions giving a completion time of approximately 15 min. With current population prospects, it is highly relevant to identify fallers among older adults before they develop physical impairments that further increase their falls risk. However, our falls prediction model needs external validation before recommendation for clinical practice.

### Implications for research

Since falls are multifactorial, they are also complex to predict. Hence, after externally validating our model, updating it with other predictors may further optimize performance. Other modeling studies have found static balance measures [14, 15] and comorbidities [11, 13, 15] to predict falls. We measured the latter but did not include this in the modeling process due to issues with implementability as diagnoses were not available for data collectors within our setting. Measures of executive and global cognitive function have also gained increased attention in falls research [41, 42]. Our study used a simple test for global cognitive function, Orientation-Memory-Concentration test, and executive function using dual tasking during balance. Similar to lower limb strength, none of these predicted falls, which could be due to the tests not differentiating well between fallers and non-fallers. After we commenced the study, recommendations for tests examining the interactions between cognition and motor function were published [43]. Here, the Montréal Cognitive Assessment [44], Trail Making Test [45], and dual-task gait measures were highlighted [43]. These tests may be more challenging for well-functioning community-dwellers, which could potentially increase the predictive performance of a model. Lastly, physical activity measures [11, 16] have been used in models, and accelerometry measures have shown promising results in falls prediction [46]. Future studies may consider updating our model using these predictors.

### Strengths and limitations

The study has a number of strengths. Missing data were sparse in the sample, the design ensured blinding within the study, and the follow-up period was long, thereby increasing chances of falls occurring. We used current recommendations on falls definitions and recording methods and reported the study transparently [20, 22]. There were also some limitations present. Half of the participants were not consecutively sampled, and the recruitment rate was low. This may have introduced selection and non-response bias that could influence predictor-outcome relationships and predictive performance when predicting falls in everyday clinical practice. This may explain the increase in MAE when performing internal validation. Furthermore, we collected predictor data within a week rather than at one point in time. This choice was a practical solution to collect data on as many predictors as possible without compromising recruitment. Predictive performance may have been influenced only to a minor degree since none of the participants fell during the first week after enrolment (data not shown), and questionnaire predictors were known to be stable over time [34, 47–50]. Furthermore, the final model was questionnaire-based, and hence predictors could be available at the time of predicting. Another limitation of the prediction model was the VES not being validated in Danish. Regarding univariate analyses, increasing age and high educational level were associated with a lower and higher falls rate, respectively. These findings are in contrast to other studies, where increasing age is associated with falls, and higher educational level is not [4]. In terms of age, this surprising finding could be due to survival bias. However, we performed a subsequent comparison between the ones dying during follow-up (*n* = 3, median age (IQR): 81 (82,83) years) and not (*n* = 238, median age (IQR): 82 (80,86) years) and found no statistically significant age difference (*p* = 0.89). Therefore, we do not believe survival bias to be of relevance in this study. However, the univariate analysis may explain this association since no adjustments for confounders were made, along with age not being included in the final model. The latter indicates that age was found less predictive of falls in the presence of other candidate predictors in this study. Regarding educational level, a comparison was made across its categories in terms of length of follow-up time, and no significant differences were found (*p* = 0.85). These findings may be due to selection bias since the same levels of education were included in the final prediction model. Overall, the results need to be confirmed in external validation studies to assess transportability to other populations.

## Conclusion

We developed a falls prediction model applicable to well-functioning community-dwelling older adults. The model consists of a time-efficient self-reported questionnaire that could add fallers’ early detection while being suitable for everyday clinical practice. The imprecision of the model was less than one fall. The results are promising, and we recommend external validation.

## Supplementary Information

**Additional file 1.** Data supplements. The data supplement provides appendices 1–4 as referenced in the manuscript text.

## Data Availability

The dataset generated and analyzed during the current study is not publicly available due to the sensitivity of personal information but is available from the corresponding author on reasonable request.

## Abbreviations

PHV: Preventive home visits
SAC: Senior activity centers
MAE: Mean absolute error
IQR: Interquartile range
CI: Confidence interval
IRR: Incidence rate ratio
TRIPOD: Transparent Reporting of a multivariable prediction model for Individual Prognosis Or Diagnosis
STROBE: Strengthening the Reporting of OBservational studies in Epidemiology
GDS4: Geriatric Depression Scale with four items
VES: Vulnerable Elders Survey 13
TFI: Tilburg Frailty Indicator
MNA: Mini Nutritional Assessment
