# Supplementary material for: Development of a multivariable prognostic PREdiction model for 1-year risk of FALLing in a cohort of community-dwelling older adults aged 75 years and above (PREFALL)
Source: BMC Geriatr. 2021 Jun 30;21:402. doi: 10.1186/s12877-021-02346-z (PMC8243769; doi:10.1186/s12877-021-02346-z)
Supplement: Supplementary file 1 — Additional file 1. Data supplements. The data supplement provides appendices 1–4 as referenced in the manuscript text. [file 12877_2021_2346_MOESM1_ESM.docx]

**Data supplements**

Table of contents

[Appendix 1: TRIPOD and STROBE checklists 2](#_Toc63243830)

[Appendix 2: study details](#_Toc63243831) 6

[Population 6](#_Toc63243832)

[Recruitment sites](#_Toc63243833) 6

[Key study dates](#_Toc63243834) 6

[Software used for data collection](#_Toc63243835) 6

[Outcome assessment](#_Toc63243836) 6

[Predictors](#_Toc63243837) 6

[Statistical analyses](#_Toc63243838) 7

[References](#_Toc63243839) 7

[Appendix 3: candidate predictors](#_Toc63243840) 8

[Appendix 4: how to calculate an individual fall rate through the model 14](#_Toc63243841)

**Appendix 1: TRIPOD and STROBE checklists**

**TRIPOD Checklist: Prediction Model Development**

| **Section/Topic** | **Item** | **Checklist Item** | **Page** |
| --- | --- | --- | --- |
| **Title and abstract** | | | |
| Title | 1 | Identify the study as developing and/or validating a multivariable prediction model, the target population, and the outcome to be predicted. | **1** |
| Abstract | 2 | Provide a summary of objectives, study design, setting, participants, sample size, predictors, outcome, statistical analysis, results, and conclusions. | **3** |
| **Introduction** | | | |
| Background and objectives | 3a | Explain the medical context (including whether diagnostic or prognostic) and rationale for developing or validating the multivariable prediction model, including references to existing models. | **5** |
|  | 3b | Specify the objectives, including whether the study describes the development or validation of the model or both. | **5** |
| **Methods** | | | |
| Source of data | 4a | Describe the study design or source of data (e.g., randomised trial, cohort, or registry data), separately for the development and validation data sets, if applicable. | **6** |
|  | 4b | Specify the key study dates, including start of accrual; end of accrual; and, if applicable, end of follow-up. | **Appendix 2** |
| Participants | 5a | Specify key elements of the study setting (e.g., primary care, secondary care, general population) including number and location of centres. | **6 + Appendix 2** |
|  | 5b | Describe eligibility criteria for participants. | **6 + Appendix 2** |
|  | 5c | Give details of treatments received, if relevant. | **Appendix 2** |
| Outcome | 6a | Clearly define the outcome that is predicted by the prediction model, including how and when assessed. | **6** |
|  | 6b | Report any actions to blind assessment of the outcome to be predicted. | **7 + Appendix 2** |
| Predictors | 7a | Clearly define all predictors used in developing or validating the multivariable prediction model, including how and when they were measured. | **7 + Appendix 3** |
|  | 7b | Report any actions to blind assessment of predictors for the outcome and other predictors. | **7** |
| Sample size | 8 | Explain how the study size was arrived at. | **7** |
| Missing data | 9 | Describe how missing data were handled (e.g., complete-case analysis, single imputation, multiple imputation) with details of any imputation method. | **8 + Appendix 2** |
| Statistical analysis methods | 10a | Describe how predictors were handled in the analyses. | **8** |
|  | 10b | Specify type of model, all model-building procedures (including any predictor selection), and method for internal validation. | **8-9** |
|  | 10d | Specify all measures used to assess model performance and, if relevant, to compare multiple models. | **8-9** |
| Risk groups | 11 | Provide details on how risk groups were created, if done. | **NA** |
| **Results** | | | |
| Participants | 13a | Describe the flow of participants through the study, including the number of participants with and without the outcome and, if applicable, a summary of the follow-up time. A diagram may be helpful. | **9-10 + Figure 1** |
|  | 13b | Describe the characteristics of the participants (basic demographics, clinical features, available predictors), including the number of participants with missing data for predictors and outcome. | **9-10** |
| Model development | 14a | Specify the number of participants and outcome events in each analysis. | **Table 3** |
|  | 14b | If done, report the unadjusted association between each candidate predictor and outcome. | **Table 2** |
| Model specification | 15a | Present the full prediction model to allow predictions for individuals (i.e., all regression coefficients, and model intercept or baseline survival at a given time point). | **Table 3** |
|  | 15b | Explain how to the use the prediction model. | **Appendix 4** |
| Model performance | 16 | Report performance measures (with CIs) for the prediction model. | **10-11+ Table 3** |
| **Discussion** | | | |
| Limitations | 18 | Discuss any limitations of the study (such as nonrepresentative sample, few events per predictor, missing data). | **11-12** |
| Interpretation | 19b | Give an overall interpretation of the results, considering objectives, limitations, and results from similar studies, and other relevant evidence. | **11-14** |
| Implications | 20 | Discuss the potential clinical use of the model and implications for future research. | **13-14** |
| **Other information** | | | |
| Supplementary information | 21 | Provide information about the availability of supplementary resources, such as study protocol, Web calculator, and data sets. | **6** |
| Funding | 22 | Give the source of funding and the role of the funders for the present study. | **15** |

**STROBE Statement—Checklist of items that should be included in reports of *cohort studies***

|  | **Item No** | **Recommendation** |  |  | **Page No.** |
| --- | --- | --- | --- | --- | --- |
| **Title and abstract** | 1 | (*a*) Indicate the study’s design with a commonly used term in the title or the abstract |  |  | 1 |
|  |  | (*b*) Provide in the abstract an informative and balanced summary of what was done and what was found |  |  | 3 |
| **Introduction** | | |  |  |  |
| Background/rationale | 2 | Explain the scientific background and rationale for the investigation being reported |  |  | 5 |
| Objectives | 3 | State specific objectives, including any pre-specified hypotheses |  |  | 5 |
| **Methods** | | |  |  |  |
| Study design | 4 | Present key elements of study design early in the paper |  |  | 6 + Appendix 2 |
| Setting | 5 | Describe the setting, locations, and relevant dates, including periods of recruitment, exposure, follow-up, and data collection |  |  | 6 |
| Participants | 6 | (*a*) Give the eligibility criteria, and the sources and methods of selection of participants. Describe methods of follow-up |  |  | 6 + Appendix 2 |
|  |  | (*b*) For matched studies, give matching criteria and number of exposed and unexposed |  |  | NA |
| Variables | 7 | Clearly define all outcomes, exposures, predictors, potential confounders, and effect modifiers. Give diagnostic criteria, if applicable |  |  | 6+ Appendix 2-3 |
| Data sources/ measurement | 8* | For each variable of interest, give sources of data and details of methods of assessment (measurement). Describe comparability of assessment methods if there is more than one group |  |  | Appendix 3 |
| Bias | 9 | Describe any efforts to address potential sources of bias |  |  | 6-7 |
| Study size | 10 | Explain how the study size was arrived at |  |  | 7 |
| Quantitative variables | 11 | Explain how quantitative variables were handled in the analyses. If applicable, describe which groupings were chosen and why |  |  | 8 + appendix 2 |
| Statistical methods | 12 | (*a*) Describe all statistical methods, including those used to control for confounding |  |  | 7-9 + appendix 2 |
|  |  | (*b*) Describe any methods used to examine subgroups and interactions |  |  | NA |
|  |  | (*c*) Explain how missing data were addressed |  |  | 8 + Appendix 2 |
|  |  | (*d*) If applicable, explain how loss to follow-up was addressed |  |  | 8-9 |
|  |  | (*e*) Describe any sensitivity analyses |  |  | Appendix 2 |
| **Results** | | |  |  |  |
| Participants | 13* | (a) Report numbers of individuals at each stage of study—eg numbers potentially eligible, examined for eligibility, confirmed eligible, included in the study, completing follow-up, and analysed |  |  | 8 + Figure 1 |
|  |  | (b) Give reasons for non-participation at each stage |  |  | Figure 1 |
|  |  | (c) Consider use of a flow diagram |  |  | Figure 1 |
| Descriptive data | 14* | (a) Give characteristics of study participants (e.g. demographic, clinical, social) and information on exposures and potential confounders |  |  | Table 1 |
|  |  | (b) Indicate number of participants with missing data for each variable of interest |  |  | Table 1 |
|  |  | (c) Summarise follow-up time (e.g., average and total amount) |  |  | 8 |
| Outcome data | 15* | Report numbers of outcome events or summary measures over time |  |  | 8 |
| Main results | 16 | (*a*) Give unadjusted estimates and, if applicable, confounder-adjusted estimates and their precision (e.g., 95% confidence interval). Make clear which confounders were adjusted for and why they were included |  |  | Table 2 |
|  |  | (*b*) Report category boundaries when continuous variables were categorised |  |  | NA |
|  |  | (*c*) If relevant, consider translating estimates of relative risk into absolute risk for a meaningful time period |  |  | NA |
| Other analyses | 17 | Report other analyses done—e.g. analyses of subgroups and interactions, and sensitivity analyses |  |  | 6 |
| **Discussion** | | |  |  |  |
| Key results | 18 | Summarise key results with reference to study objectives |  |  | 10 |
| Limitations | 19 | Discuss limitations of the study, taking into account sources of potential bias or imprecision. Discuss both direction and magnitude of any potential bias |  |  | 11-12 |
| Interpretation | 20 | Give a cautious overall interpretation of results considering objectives, limitations, multiplicity of analyses, results from similar studies, and other relevant evidence |  |  | 11-14 |
| Generalisability | 21 | Discuss the generalisability (external validity) of the study results |  |  | 12 |
| **Other information** | | |  |  |  |
| Funding | 22 | Give the source of funding and the role of the funders for the present study and, if applicable, for the original study on which the present article is based |  |  | 15 |

#

**Appendix 2: study details**

**Population**

For all 75+-year olds in the municipality, the median age (IQR) was 80 years (77 – 85) which is similar to national figures of 80 (77-84) years (1). The mean life expectancy was 81.1 years which was slightly higher than a national level of 80.5 years (2).

**Recruitment sites**

According to Danish legislation on health and social services, Danish municipalities are responsible for developing and initiating prophylactic and health-promoting initiatives for their senior citizens. This is done through different authorities in the municipality (e.g. preventive-home-visits (PHV) or senior activity centres (SAC))(3). Thus, recruitment for the study was performed through these initiatives. For PHVs, the target group is all community-dwelling older adults, primarily at the age of 75 years and above within the municipality. Furthermore, data collection was performed by trained nurses who recruited participants through a consecutive sampling strategy along with performing baseline data collection in participants own homes. In 2019, 1,794 (28.9%) older adults of the accessible population received a PHV (4).

Regarding SACs, in 2018, 318 citizens attended these weekly within the municipality. Here, healthcare workers collected baseline data and recruited participants through a convenient sampling strategy. A consecutive sampling method was not possible due to SACs being public facilities with varying attendees each day. Three SACs were involved in the study located in the cities of Hjørring, Sindal, and Hirtshals. This was chosen due to economic and administrative reasons. Lastly, participants were also conveniently recruited by presenting the study at gatherings in senior clubs and associations within the municipality. To minimise non-response bias, recruitment videos were transmitted by the local televisions channel along with ads in newspapers and social media. The first author performed baseline data collection in this recruitment site.

**Study dates**

The start of recruitment and follow-up was June 14, 2018. Recruitment ended on July 18, 2019, and follow-up ended on July 18, 2020.

**Software used for data collection**

Study data were collected and managed using REDCap electronic data capture tools hosted at Region Nordjylland (5).

**Outcome assessment**

If a calendar was not received, a trained research secretary phoned participants asking them to send the calendar to minimise loss to follow-up. Furthermore, if a fall was recorded in the calendar, the secretary called to verify whether the fall fitted the outcome definition and recorded further details. These procedures were similar for all participants. The first author performed data quality checks through unannounced inspections throughout the study to check whether the outcome assessors used the correct fall definition and recording methods during follow-up. The last author verified deaths through hospital records. All outcome assessors were blinded towards baseline predictors by not having access to these data in REDCap.

**Predictors**

Hardware for measuring balance, strength, reaction time was recalibrated for each new participant. Furthermore, the first author recorded diagnoses and prescribed drugs from hospital records. However, diagnoses were not used as candidate predictors, but only for describing the sample since these were not available for data collectors within our setting. Furthermore, medication was summarised into the number of prescribed drugs since this would be possible within our setting.

Before commencing recruitment, data collectors were provided with a training session led by the first and second author. Manuals were provided to ensure predictors were defined and assessed in a similar way for all participants. Furthermore, data collectors were not trained to interpret test results but only perform the tests to avoid participants changing their behaviour and potentially the falls risk due to baseline tests results. The first author made unannounced inspections to check whether data collection methods were in line with the study protocol.

Physical tests were performed on the inclusion date, after which a self-report questionnaire was handed out as paper or electronically. Within two days from the inclusion date, the first author recorded diagnoses and prescribed medication from hospital records. Within a week from the inclusion date, the first author phoned participants to do the cognitive tests and record questionnaire answers to reduce the potential for missing data. However, as a precaution to minimise bias due to contact between the first author and participants, participants were asked to fill out the questionnaire before the first author recorded the answers.

In PHVs, which generally last one hour per older adult, the study was granted 35 minutes for baseline data collection. Since PHVs aim to preserve older adults’ functional capacities, tests were performed after the nurses had completed their usual duties for the first 25 minutes of the visit. Thus, supporting recommendations for the older adults would not be based on test results but regular clinical practice. We chose not to include the supporting recommendation as candidate predictors in the model building process since these were too poorly defined.

**Statistical analyses**

*Comparison of recruitment sites and follow-up completeness*

For continuous variables, the three recruitment sites were analysed by the Likelihood ratio test. Furthermore, a comparison between participants lost to follow-up and those retained in the study was performed on demographic characteristics. Here, Wilcoxon rank sum test was used for continuous variables. For both comparisons, the chi-squared test was performed to compare categorical variables when the expected number of observations in each cell was higher than five. Otherwise, Fischer’s Exact test was used.

*Missing data*

The strategy on mode imputation in categorical variables was based on sensitivity analyses where less than ten observations in a level of a categorical variable would lead to missing levels in either training or tests datasets when using bootstrap validation and random forest imputation.

**Results:**

In terms of comparison between recruitment sites, median (IQR) ages were 85 (80.8-89) years for SACs, 82 (80-86) years for PHVs, and 80 (76-83) years for senior clubs (p = 0.0007). Furthermore, 41/56 (73%) SAC participants had completed primary school as the highest level of education compared with 42/127 (33%) in PHVs and 23/58 (40%) in senior clubs (*p* < 0.001).

**References**:

1. Danmarks statistik. Folketal den 1. i kvartalet efter område, alder og tid. https://www.statistikbanken.dk/folk1a.

2. Danmarks statistik. Middellevetid for 0-årige efter område og tid. https://www.statistikbanken.dk/hisbk.

3. Sundhedsstyrelsen. *Forebyggelse På Ældreområdet - Håndbog Til Kommunerne*.; 2015. https://www.sst.dk/-/media/Udgivelser/2015/Forebyggelse-på-ældreområdet---håndbog-til-kommunerne/Forebyggelse-på-ældreområdet,-d-,-Håndbog-til-kommunerne.ashx?la=da&hash=74B7EC7B72EA05AD8BE434F866BFA586AC36BF9B.

4. Danmarks statistik. Modtagere af forebyggende hjemmebesøg, efter område, hjemmebesøg, alder og køn. https://www.statistikbanken.dk/aed10a.

5. Harris PA, Taylor R, Thielke R, Payne J, Gonzalez N, Conde JG. Research electronic data capture (REDCap)-A metadata-driven methodology and workflow process for providing translational research informatics support. *J Biomed Inform*. 2009;42(2):377-381. doi:10.1016/j.jbi.2008.08.010.

**Appendix 3: eTable 1: candidate predictors**

| **Number** | **Candidate predictor variable** | **Obtained through:**  **Physical tests (T) at baseline or Self-report questionnaire (Q) within one week from baseline via a telephone call** | **Variable type** | **Degrees of freedom^a^** | **Response options or units** | **Summary measure** | **Distribution** | **Expected effect on absolute risk**  **+ =** increases risk  - = decreases risk  ? = unknown effect or effect may be both + and - |
| --- | --- | --- | --- | --- | --- | --- | --- | --- |
| 1 | Age | Q | Continuous | 1 | Years | median [IQR] | 82 [80, 86] | + |
| 2 | Sex | Q | Categorical | 1 | 1. Men 2. Women | n (%) | 1. 81 (33.6) 2. 160 (66.4) | + for women |
| 3 | Educational level | Q | Categorical | 5 | 1. Municipal primary and lower secondary school 2. General upper secondary education 3. Skilled worker 4. Short-cycle higher education 5. Medium-cycle higher education 6. Long cycle higher education | n (%) | 1. 106 (44.0) 2. 8 (3.3) 3. 68 (28.2) 4. 12 (5.0) 5. 39 (16.2) 6. 8 (3.3) | ? |
| 4 | Marital status | Q | Categorical | 3 | 1. Married/living with a partner 2. Unmarried 3. Separated/divorced 4. Widow/widower | n (%) | 1. 98 (40.7) 2. 8 (3.3) 3. 10 (4.1) 4. 125 (51.9) | ? |
| 5 | Living alone | Q | Categorical | 1 | 1. Yes 2. No | n (%) | 1. 144 (59.8) 2. 97 (40.2) | + for “Yes” |
| 7 | Prior falls | Q | Categorical | 1 | 1. None 2. One or more falls within the last year | n (%) | 1. 136 (56.4) 2. 105 (43.6) | + for prior falls |

| **Number** | **Candidate predictor variable** | **Obtained through:**  **Physical tests (T) at baseline or Self-report questionnaire (Q) within one week from baseline via a telephone call** | **Variable type** | **Degrees of freedom^a^** | 1. **Response options or units** | **Summary measure** | 1. **Distribution** | **Expected effect on absolute risk**  **+ =** increases risk  - = decreases risk  ? = unknown effect or effect may be both + and - |
| --- | --- | --- | --- | --- | --- | --- | --- | --- |
| 8 | Two or more diseases and/or chronic conditions | Q | Categorical | 1 | 1. Yes 2. No | n (%) | 1. 209 (86.7) 2. 32 (13.3) | + for” Yes” |
| 9 | Medication | Q | Continuous | 1 | Number of drugs prescribed | median [IQR] | 7 [4, 10] | + |
| 10 | Use of assistive devices | Q | Categorical | 1 | 1. Yes 2. No | n (%) | 1. 142 (58.9) 2. 99 (41.1) | + for” Yes” |
| 11 | Weekly alcohol consumption | Q | Categorical | 3 | 1. Less than seven units weekly 2. 7-14 units weekly 3. 15-21 units weekly 4. more than 21 units weekly | n (%) | 1. 187 (77.6) 2. 47 (19.5) 3. 5 (2.1) 4. 2 (0.8) | + |
| 12 | Using multifocal lenses | Q | Categorical | 1 | 1. Yes 2. No | n (%) | 1. 184 (76.3) 2. 57 (23.7) | + for” Yes” |
| 13 | Having dogs or cats in the household | Q | Categorical | 1 | 1. Yes 2. No | n (%) | 1. 30 (12.4) 2. 211 (87.6) | ? |
| 14 | Health-related quality of life (EQ-5D-3L by the EuroQol group) | Q | Continuous | 1 | Score from -0.167 to 1.000 points | Median [IQR] | 0.7 [0.6, 1.0] | ? |

| **Number** | **Candidate predictor variable** | **Obtained through:**  **Physical tests (T) at baseline or Self-report questionnaire (Q) within one week from baseline via a telephone call** | **Variable type** | **Degrees of freedom^a^** | **Response options or units** | **Summary measure** | **Distribution** | **Expected effect on absolute risk**  **+ =** increases risk  - = decreases risk  ? = unknown effect or effect may be both + and - |
| --- | --- | --- | --- | --- | --- | --- | --- | --- |
| 15 | Nutritional status (Mini Nutritional Assessment by Nestle) | Q | Continuous | 1 | Score from 0-14 point (14 being the best possible score obtained) | mean (SD) | 11.1 (1.7)  Missing: n = 1 | - |
| 16 | Occasionally experiencing urinary incontinence | Q | Categorical | 1 | 1. Yes 2. No | n (%) | 1. 90 (37.3) 2. 151 (62.7) | + for” Yes” |
| 17 | Occasionally experiencing lower limb pain when walking | Q | Categorical | 1 | 1. Yes 2. No | n (%) | 1. 121 (50.2) 2. 120 (49.8) | + for” Yes” |
| 18 | Occasionally experiencing dizziness | Q | Categorical | 1 | 1. Yes 2. No | n (%) | 1. 123 (51.0) 2. 118 (49.0) | + for” Yes” |
| 19 | Do you think you will fall within the next year? | Q | Categorical | 1 | 1. Yes 2. No | n (%) | 1. 45 (18.8) 2. 194 (81.2)   Missing: n = 2 | ? |
| 20 | Fear of falling (Short Falls Efficacy Scale International - 7 items) | Q | Continuous | 1 | Score from 7-28 points (7 is the best possible score) | median [IQR] | 8 [7, 11] | + |

| **Number** | **Candidate predictor variable** | **Obtained through:**  **Physical tests (T) at baseline or Self-report questionnaire (Q) within one week from baseline via a telephone call** | **Variable type** | **Degrees of freedom^a^** | **Response options or units** | **Summary measure** | 1. **Distribution** | **Expected effect on absolute risk**  **+ =** increases risk  - = decreases risk  ? = unknown effect or effect may be both + and - |
| --- | --- | --- | --- | --- | --- | --- | --- | --- |
| 21 | Depressive symptoms using the Geriatric Depression Scale (4 items) | Q | Categorical | 1 | Score from 0-4 points (0 is the best possible score)   1. 0 points 2. 1 point 3. 2 points 4. 3 points 5. 4 points | n (%) | 1. 173 (71.8) 2. 55 (22.8) 3. 11 (4.6) 4. 2 (0.8) 5. 0 (0) | + |
| 22 | Frailty (Tilburg Frailty Indicator - Part B) | Q | Continuous | 1 | Score from 0-15 points (0 is the best possible score) | Median [IQR] | 6 [4, 8]  Missing: n = 1 | + |
| 23 | Activities of Daily Living (Vulnerable Elders Survey) | Q | Continuous | 1 | Score from 1-10 points (1 is the best possible score) | Median [IQR] | 3 [1, 4]  Missing: n = 4 | + |
| 24 | Orientation-Memory-Concentration | Q | Continuous | 1 | Score from 0-28 points (28 is the best possible score). | Median [IQR] | 26 [22, 26]  Missing: n = 4 | - |
| 25 | Average both lower limbs’ reaction time | T: Fysiometer | Continuous | 1 | Milliseconds | Median [IQR] | 1,315 [1,122, 1,567]  Missing: n= 2 | + |
| 26 | Difference in both lower limbs’ reaction time | T: Fysiometer | Continuous | 1 | Milliseconds | Median [IQR] | 142 [60, 370]  Missing: n = 2 | + |

| **Number** | **Candidate predictor variable** | **Obtained through:**  **Physical tests (T) at baseline or Self-report questionnaire (Q) within one week from baseline via a telephone call** | **Variable type** | **Degrees of freedom^a^** | **Response options or units** | **Summary measure** | **Distribution** | **Expected effect on absolute risk**  **+ =** increases risk  - = decreases risk  ? = unknown effect or effect may be both + and - |
| --- | --- | --- | --- | --- | --- | --- | --- | --- |
| 27 | Total isometric strength of both lower limbs | T: Fysiometer | Continuous | 1 | Kilogram | Mean (SD) | 146.5 (75.5)  Missing: n = 2 | - |
| 28 | Difference isometric strength of between lower limbs | T: Fysiometer | Continuous | 1 | Kilogram | Median [IQR] | 7.1 [3.4, 11.9]  Missing: n= 2 | + |
| 29 | Total grip strength of both upper limbs | T: Fysiometer | Continuous | 1 | Kilogram | Mean (SD) | 25.5 (9.6)  Missing: n = 2 | - |
| 30 | Difference in grip strength between upper limbs | T: Fysiometer | Continuous | 1 | Kilogram | Median [IQR] | 1.5 [0.7, 2.5]  Missing: n = 2 | ? |
| 31 | Balance with dual tasking - Centre of Pressure speed | T: Fysiometer | Continuous | 1 | Speed in millimetres/second | Median [IQR] | 23 [16, 30]  Missing: n = 1 | ? |
| 32 | Balance with dual tasking - Centre of Pressure area | T: Fysiometer | Continuous | 1 | Sway area in mm^2^ | Median [IQR] | 69 [42, 112]  Missing: n = 1 | ? |

| **Number** | **Candidate predictor variable** | **Obtained through:**  **Physical tests (T) at baseline or Self-report questionnaire (Q) within one week from baseline via a telephone call** | **Variable type** | **Degrees of freedom^a^** | **Response options or units** | **Summary measure** | **Distribution** | **Expected effect on absolute risk**  **+ =** increases risk  - = decreases risk  ? = unknown effect or effect may be both + and - |
| --- | --- | --- | --- | --- | --- | --- | --- | --- |
| 33 | Habitual gait speed (4 m) | T: Timer and measured out distance in homes | Continuous | 1 | Metres/second | Median [IQR] | 0.9 [0.7, 1.2] | - |
| 34 | Body Mass Index | T: Derived from weight and height measures | Continuous | 1 | Kilograms/metres^2^ | Mean (SD) | 28.3 (4.7)  Missing: n = 1 | ? |

*Notes:* ^a^ = Total number of degrees of freedom = 41

**Appendix 4: eTable 2: how to calculate an individual fall rate through the model**

For calculating the fall rate, this section will provide an example using a fictitious case of a community-dwelling woman at the age of 87 years. She is a former nurse. She has fallen within the last year and believes she will fall again within the next year. She drinks between 7-14 units of alcohol weekly and occasionally experiences dizziness. When asking her to complete the questionnaires for Vulnerable Elders Survey 13 and the 4-item Geriatric Depression Scale, she receives a score of 6 and 0 points, respectively.

When applying this information in the model, the older adult receives a predicted fall rate of about three falls per year. Taking the model’s mean absolute error of ± 0.88 falls into account, the older adult is predicted to fall at least two times during one year.

| **Predictors** | **β-coefficients** | **Response:**  **No = 0, Yes = 1,**  **or score** | **Linear predictor** |
| --- | --- | --- | --- |
| Intercept | -6.89417948 | - | -6.89417948 |
| Short-cycle higher education (completed) | 0.14752490 | 0 | 0 |
| Medium-cycle higher education | 0.22365429 | 1 | 0.22365429 |
| Occasional dizziness (present) | 0.17787573 | 1 | 0.17787573 |
| Weekly alcohol consumption: 7-14 units (yes) | 0.28918905 | 1 | 0.28918905 |
| Fallen within the last year (yes) | 0.50322396 | 1 | 0.50322396 |
| Self-perceived falls risk (will fall) | 0.74611041 | 1 | 0.74611041 |
| VES-score, per point | 0.01768701 | 6 | 0.10612206 |
| 3 points on GDS4 | 1.21255321 | 0 | 0 |
|  | | | |
| **Sum of linear predictors** | | | -4.84800398 |
|  | | | |
| **Description** | **Formula** | | **Number of falls** |
| Fall rate per year | exp^(-4.84800398+ ln(365)^ | | 2.86306684 |
| *Notes:* VES = Vulnerable Elders Survey, GDS4 = Geriatric Depression Scale with 4 items, kg = kilogram, Ln = natural logarithm, exp = exponential | | | |
